# Approaches to Evaluate Whole Exome Sequencing Data That Incorporate Genetic Intolerance Scores for Congenital Anomalies, Including Intronic Regions Adjacent to Exons

**DOI:** 10.1002/mgg3.70092

**Published:** 2025-03-13

**Authors:** Kosuke Taniguchi, Fuyuki Hasegawa, Yuka Okazaki, Asuka Hori, Hiroko Ogata‐Kawata, Saki Aoto, Ohsuke Migita, Tomoko Kawai, Kazuhiko Nakabayashi, Kohji Okamura, Kana Fukui, Seiji Wada, Katsusuke Ozawa, Yushi Ito, Haruhiko Sago, Kenichiro Hata

**Affiliations:** ^1^ Department of Maternal‐Fetal Biology National Research Institute for Child Health and Development Tokyo Japan; ^2^ Department of Human Molecular Genetics Gunma University Graduate School of Medicine Maebashi Gunma Japan; ^3^ Center for Genetic Medicine National Center for Child Health and Development Tokyo Japan; ^4^ Center for Maternal‐Fetal, Neonatal and Reproductive Medicine National Center for Child Health and Development Tokyo Japan; ^5^ Department of Nursing Nippon Medical School Musashikosugi Hospital Kanagawa Japan; ^6^ Medical Genome Center National Research Institute for Child Health and Development Tokyo Japan; ^7^ Department of Laboratory Medicine St. Marianna University, School of Medicine Kanagawa Japan; ^8^ Department of Systems Biomedicine National Center for Child Health and Development Tokyo Japan

**Keywords:** congenital anomalies, exons, genetic intolerance scores, intronic regions, whole exome sequencing

## Abstract

**Background:**

Whole exome sequencing (WES) aids in diagnosing monogenic diseases, yet > 50% of all cases remain undiagnosed. We aimed to improve diagnostic precision by developing an effective WES‐based strategy for detecting congenital anomalies.

**Methods:**

Initially, 128 probands with congenital anomalies were assessed using trio‐WES and copy number variation analysis—variant interpretation was for exons and splice sites. Thereafter, we reanalyzed the sequence data for undiagnosed cases using the following methods. First, we performed trio‐WES analysis, adding genetic intolerance scores annotation. Second, we analyzed all exons, splicing sites, and intron variants for cases with phenotypes suggestive of specific causative genes using SpliceAI. Lastly, using SpliceAI, we analyzed all exons, splicing sites, and intron variants in genetically constrained genes filtered with genetic intolerance scores.

**Results:**

Initial analysis diagnosed 51 of 128 cases (39.8%). In the reanalysis, first, we identified novel likely pathogenic variants in *MED12* and *CCDC22* associated with X‐linked diseases. Second, a novel *TMEM67* intron variant associated with Meckel syndrome was detected. Finally, a de novo hemizygous pathogenic intronic variant in *CASK* was identified in a case of intrauterine fetal death.

**Conclusions:**

WES analysis, including intronic regions and utilizing genetic intolerance scores, has the potential to efficiently improve diagnostic yield.

## Graphical abstract

Evaluation of variants, including introns, in combination with the genetic intolerance score, is expected to improve the diagnostic yield of WES for congenital anomalies.

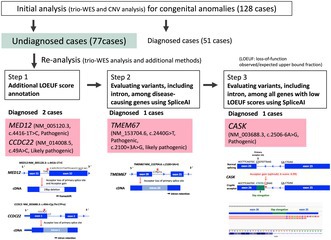

## 1 Introduction

The introduction of whole exome sequencing (WES) has resulted in substantial advancement in the field of prenatal diagnostics (Lord et al. 2019; Mellis et al. 2022; Petrovski et al. 2019; Sparks et al. 2020; Stanley et al. 2020). Ultrasonography remains the cornerstone for detecting congenital anomalies owing to its cost‐effectiveness and ease of use (Hunter and Simpson 2014). Accurate diagnosis of these anomalies is pivotal in determining the prognosis, devising therapeutic interventions during pregnancy or postpregnancy, and assessing the risk of recurrence during subsequent pregnancies (Lord et al. 2019). On the contrary, the diagnostic rate with WES ranges from 5% to 37% for diverse congenital anomalies (Mellis et al. 2022). This figure may reflect the inclusion of cases with nonmonogenic etiologies and the possibility that some genetic causes have not yet been identified owing to the current limitations of standard WES diagnostic methodologies. To solve this problem, a comprehensive analysis and variant evaluation of WES data using the latest tools have emerged as a feasible solution to improve diagnostic outcomes (Salfati et al. 2019).

Recently, as genomic data from the general population have become more robust, accurate genetic intolerance scores based on statistical evaluations of molecular evolution in genes have emerged (Karczewski et al. 2020). The loss‐of‐function (LOF) observed/expected upper bound fraction (LOEUF) score facilitates the identification of genes with fewer LOF variants than expected in the general population. This serves as a metric to gauge a gene's intolerance to LOF mutations (Petrovski et al. 2013; Karczewski et al. 2020). Deficiency in genes with low LOEUF scores can lead to haploinsufficiency. Thus, the LOEUF score may be pivotal for assessing disease‐causative genes. A recent study on stillbirth cohorts using WES revealed LOF variant enrichment in genes with low LOEUF scores among stillbirths (Stanley et al. 2020). This finding indicates that gene evaluation using LOEUF scores could be beneficial for variant filtering in cases with complex phenotypes, such as multiple congenital anomalies, where identifying the causative gene may be challenging.

A recent study showed that WES data includes meaningful variants in intronic regions adjacent to exons and highlighted the value of incorporating intronic variant analysis into WES data interpretation. Moreover, modern tools leveraging deep learning, such as SpliceAI (Jaganathan et al. 2019) and Pangolin (Zeng and Li 2022), which can identify mutations that hinder splicing or generate aberrant isoforms, have been effectively used to diagnose rare diseases (Lord and Baralle 2021; Setty et al. 2022). Therefore, we reanalyzed WES data from previously undiagnosed cases in our trio‐WES analyses for congenital anomalies. Here, we first present the diagnostic yield from the initial analysis, followed by an investigation into whether incorporating genetic intolerance scores and variant evaluation including intronic regions in our reanalysis could aid in the diagnosis of these anomalies.

## 2 Materials and Methods

### 2.1 Participants

**FIGURE 1:**
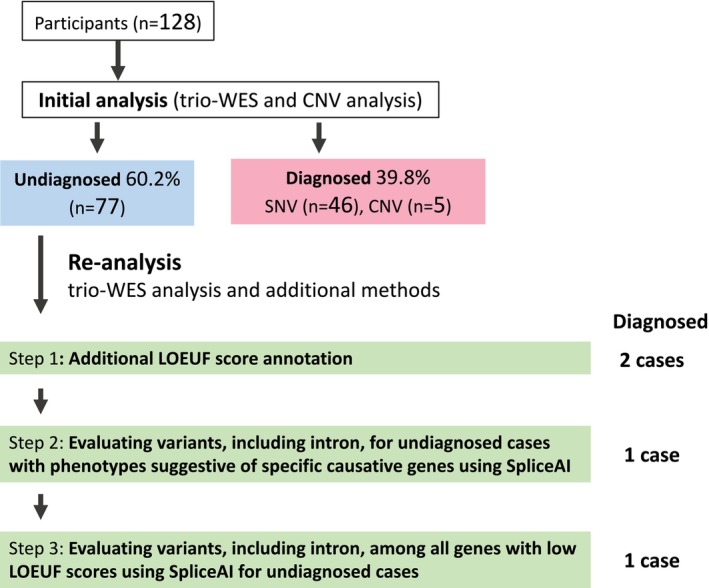
Flow chart depicting the diagnosis. CNV, copy number variation; LOEUF, loss‐of‐function observed/expected upper bound fraction; WES, whole exome sequencing.

Cases wherein congenital anomalies were identified using prenatal ultrasonography initially were subjected to trio‐WES between 2011 and 2021 at the National Centre for Child Health and Development (NCCHD) in Tokyo, Japan (Figure 1 and Table S1). Trio‐WES was performed after comprehensive discussions between fetal imaging specialists and clinical genetic experts. Specimens were collected from probands, patients, and, in some cases, siblings in the study. Cases with evident chromosomal abnormalities or congenital anomalies caused by known infections were excluded. Furthermore, probands solely presenting with increased nuchal translucency were excluded. This study was approved by the Institutional Review Board (IRB) of NCCHD (IRB number: 236,926). Written informed consent was obtained from all participants or their legal representatives.

### 2.2 Disease Type Definition and Classification

Multiple malformations were classified as structural abnormalities in more than one organ. Skeletal dysplasia and abnormalities of the cardiovascular, central nervous, abdominal wall, joint, chest/respiratory, lymphatic, and renal systems were classified as the main structural abnormalities of various organ systems. Hydrops fetalis was defined as an abnormal collection of fluid in ≥ 2 areas of the body in a fetus with no structural anomalies. Fetal pleural effusion necessitating surgical intervention was defined as effusion.

### 2.3 Whole Exome Sequencing (WES)

Genomic DNA was collected from cord blood, peripheral blood, umbilical cord, placenta, amniotic fluid cells, and saliva. WES libraries were prepared using a SureSelect Capture Kit (Agilent Technologies, CA, USA) in accordance with the manufacturer's instructions. A HiSeq 2500 (Illumina, CA, USA) or a DNBSEQ‐G400 BGI sequencer (BGI, Shenzhen, China) was used to sequence the libraries (Table S2).

### 2.4 Initial Analysis

In the initial analysis, trio‐WES analysis focused on candidate variants targeting the exon regions and splice sites (±2 bp positions from the exon–intron boundary) with inheritance filtering. Details of the WES analysis pipeline and variant filtering flow are provided in Table S2. Additionally, the Infinium Asian Screening Array‐24 v1.0 BeadChip Kit (Illumina) and EXCAVATOR 2 v1.1.2 (a WES‐based copy number variation (CNV) detection tool) were used to perform CNV analysis. Disease causal variants classified as likely pathogenic or pathogenic were selected according to the American College of Medical Genetics and Association for Molecular Pathology (ACMG/AMP) variant interpretation guidelines (Richards et al. 2015).

### 2.5 Reanalysis

The reanalysis included trio‐WES analysis and additional methods for genetically undiagnosed cases (Figure 1). In the reanalysis, we analyzed the trio‐WES data from the raw sequence data using updated software and databases, focusing on candidate variants in the exons and splice sites. Additionally, we incorporated the following three additional methods. Details are provided in Table S2. InterVar (an online platform that adheres to the ACMG/AMP 2015 variant interpretation guidelines) was used to interpret the variants (Li and Wang 2017).

Step 1: We added the LOEUF score as an annotation to the WES analysis results as a genetic intolerance score. Genes with LOEUF scores below 0.35 may cause haploinsufficiency; thus, we particularly focused on autosomal dominant (AD) and X‐linked genes.

Step 2: For undiagnosed cases with a clinical diagnosis (see below) where the causative gene could be inferred, all exon, splice site, and intron variants for disease‐specific genes were evaluated (Figure 1). Disease‐specific genes of hydrops fetalis, effusions, skeletal disorders, Joubert syndrome, and Meckel syndrome are described in Table S3. After preparing the gene lists, SpliceAI analysis was automated using the Bash and Python scripts with trio‐Variant Call Format (VCF) files as input to evaluate splicing effects for each variant, including the screening of candidates with SpliceAI scores (delta score > 0.2) (the automated script execution time was approximately 5–10 min per family).

Step 3: Exon, splice site, and intron variants on genes with low LOEUF scores (2971 genes) were evaluated with SpliceAI, automated on the Bash and Python scripts using trio‐VCF as input, for undiagnosed cases after Steps 1 and 2. During this process, candidate variants were also automatically screened based on variant frequency and SpliceAI score (delta score > 0.2), focusing primarily on those associated with de novo AD and X‐linked disorders (the automated script execution time was approximately 30–60 min per family).

In all cases, inheritance filtering was applied during variant evaluation (Steps 1–3).

In Steps 2 and 3, reanalysis was performed for candidate genes, including up to an average of 150 bp from the exon–intron boundary.

### 2.6 RNA Sequencing

The RNeasy Fibrous Tissue Mini Kit (Qiagen, Hilden, Germany) was used to extract total RNA from the placental villi of cases and normal controls. The RNase‐Free DNase Set (Qiagen) was used to remove genomic DNA, and the NEBNext Ultra II Directional RNA Library Prep Kit for Illumina (New England Biolabs, Ipswich, MA, USA) was used to create the RNA sequence library. Sequencing was performed using NovaSeq6000 (Illumina) with a 100 bp paired‐end mode. HISAT2 software was used for mapping. Integrative Genomics Viewer (IGV) 2.5.2 was used to visualize the results (Robinson et al. 2011).

### 2.7 Loss‐of‐Function Observed/Expected Upper Bound Fraction Score Comparison Across Online Mendelian Inheritance in Man Database Genes

Genes with LOEUF scores were selected from the 17,425 genes listed in the Online Mendelian Inheritance in Man (OMIM) database divided into autosomes and X chromosomes. The LOEUF scores were available for 13,716 and 593 autosomal and X‐chromosomal genes, respectively. The phenotypic information registered in the OMIM database was used to classify these genes based on the inheritance form of the disease. The LOEUF scores were compared for each category (Table S4).

### 2.8 Statistical Analyses

The LOEUF scores were compared using Welch's *t* test. All statistical analyses were performed using R v4.1.0 (R Foundation for Statistical Computing, Vienna, Austria).

## 3 Results

### 3.1 Diagnostic Results of the Initial Analysis

**FIGURE 2:**
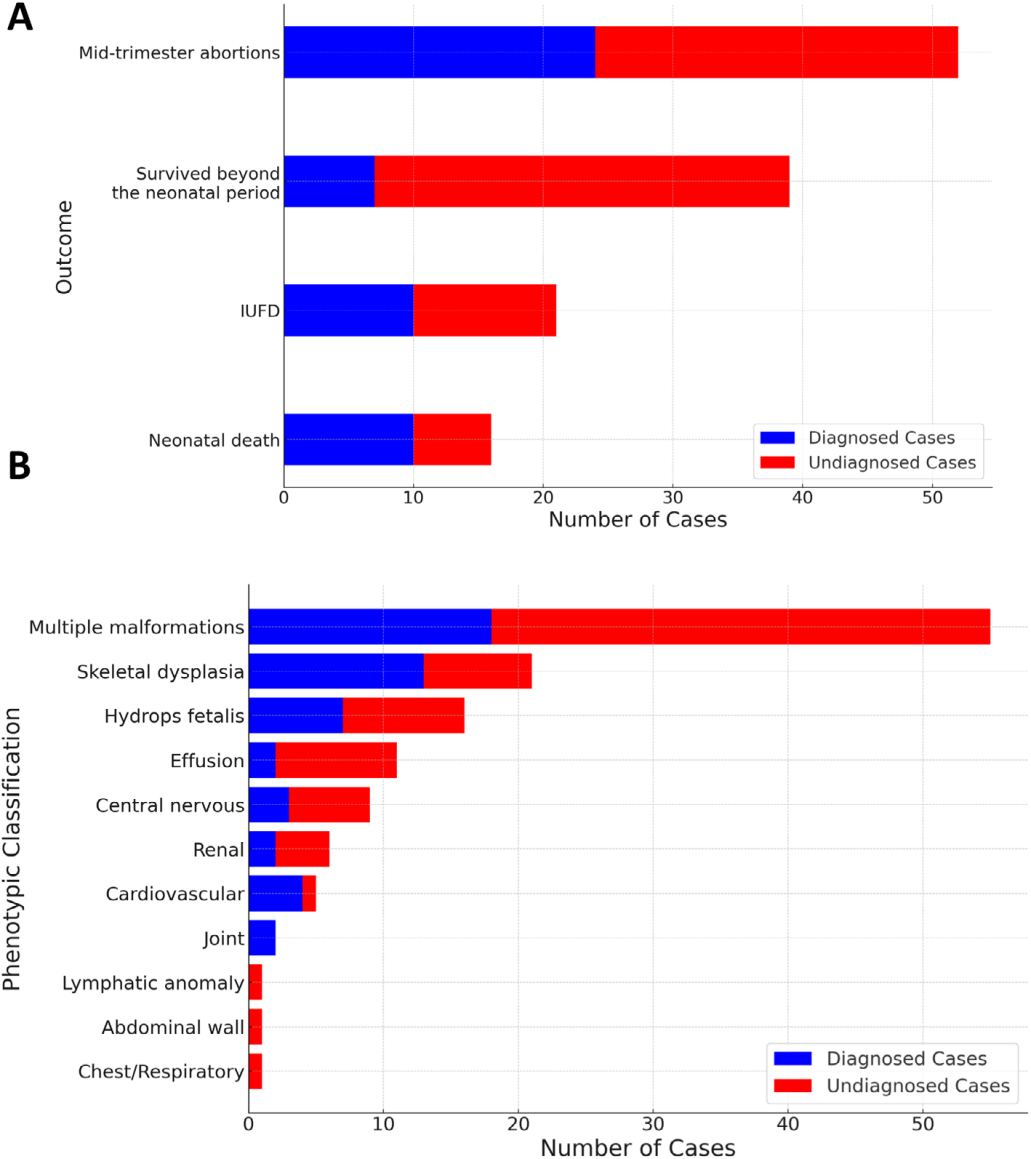
Breakdown of cases during initial analysis. (A) Breakdown of cases based on clinical outcome. (B) Breakdown of cases based on phenotypic category.

A total of 128 patients with congenital anomalies underwent trio‐WES and CNV analysis. Figure 1 presents the diagnostic flowchart. Initial analysis identified the causative pathogenic variants [single nucleotide variations (SNVs) or CNVs] in 51 of the 128 cases, corresponding to a diagnostic yield of 39.8%. Neonatal deaths were associated with a higher diagnostic rate (Figure 2). Moreover, an elevated diagnostic yield was observed for joint abnormalities, cardiovascular abnormalities, and skeletal dysplasia (Figure 2). A summary of phenotypes and WES results for all cases is presented in Table S1.

### 3.2 Reanalysis for Undiagnosed Cases

**FIGURE 3:**
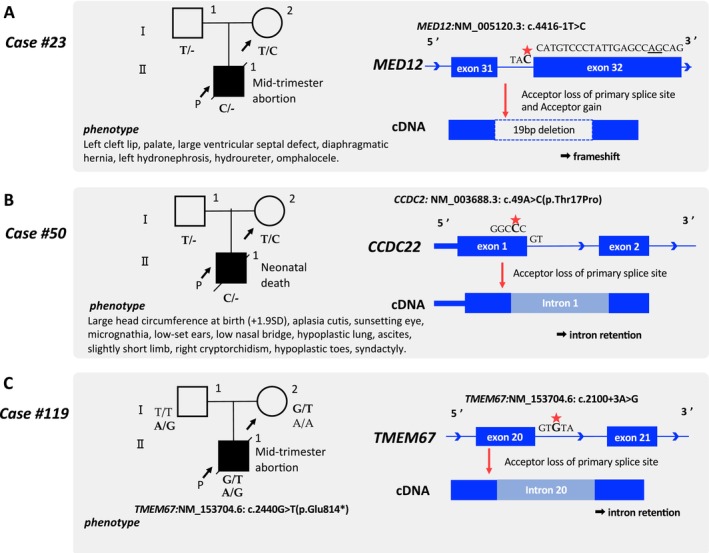
Details of cases where pathogenic variants were identified in reanalysis during Steps 1 and 2. A, B, and C show pedigree, phenotype, genotype, and overview of the intronic variant leading to aberrant splicing in each case during Steps 1 and 2 (A: Case #23, B: Case #50, C: Case #119).

**FIGURE 4:**
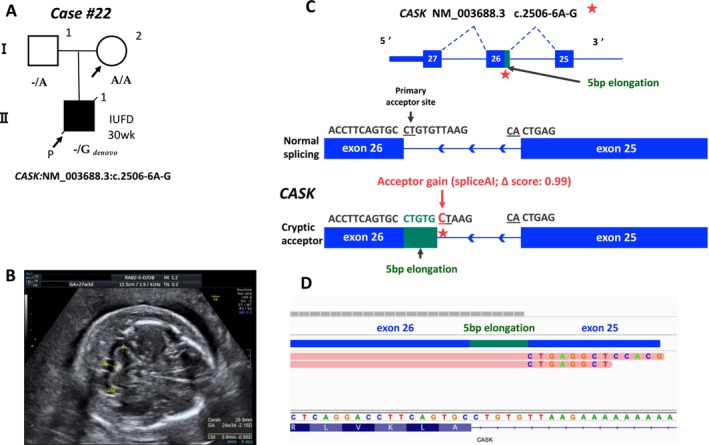
Details of the CASK‐related disorder case detected during Step 3. (A) Pedigree of this case and each genotype. (B) Ultrasound data for severe subcutaneous edema and mild cerebellar hypoplasia in utero at gestational week 27. (C) Overview of the intronic variant leading to aberrant splicing. (D) RNA sequencing showed aberrant splicing on CASK.

In the reanalysis, we could identify the causative pathogenic variants in four additional cases, incorporating the additional methods (Figures 1, 3, and 4). The pathogenic variants detected through reanalysis may have affected splicing abnormalities (Table 1). The cases diagnosed at each step are presented below.

**TABLE 1:** Details of newly identified pathogenic and likely pathogenic variants.

Case #	Gene variant information	MAF on gnomAD	SpliceAI Δtype:Δscore (pre‐mRNA position)	Pangolin Δtype:Δscore (pre‐mRNA position)	MaxEntScan score using the maximum entropy model	Other information
24	*MED12* NM_005120.3:c.4416‐1 T>C	0	Acceptor loss: 0.97 (1 bp) Acceptor gain: 0.80 (20 bp)	Splice loss: 0.82 (1 bp) Splice gain: 0.63 (20 bp)	Variation 9.5 > 1.43 (−84.95%)	
54	*CCDC22* NM_003688.3: c.49A>C, (p.Thr17Pro)	0	Donor loss: 0.05 (1 bp)	Splice Loss: 0.22 (1 bp)	Variation 7.47 > 5.46 (−26.9%)	CADD PHRED: 29.7, SIFT (score 0.08: damaging), PolyPhen‐2 (score 0.99: deleterious)
129	*TMEM67* NM_153704.6: c.2100 + 3A>G	0	Donor loss: 0.38 (−3 bp)	Splice loss: 0.37 (−3 bp)	Variation 5.96 > −1.06 (−117.8%)	
23	*CASK* NM_003688.3: c.2506‐6A‐G	0	Acceptor loss: 0.13 (−6 bp) Acceptor gain: 0.9 (−1 bp)	Splice Loss: 0.69 (−6 bp) Splice gain: 0.84 (−1 bp)	Variation 7.77 > 3.32 (−57.3%)	

Abbreviations: CDH, congenital diaphragmatic hernia; MAF, minor allele frequency; MaxEntScan, maximum entropy modeling of short sequence motifs; VSD: ventricular septal defect.

### 3.3 Step 1: Additional LOEUF Score Annotation

We assessed all variants in exons and splicing sites, adding LOEUF score annotations, for 77 cases that remained undiagnosed even after the initial analysis (Figure 1). In Case #23, multiple malformations were observed in the proband (Figure 3A). Although the SNP array indicated a normal female karyotype, the pregnancy was terminated mid‐term. A novel de novo hemizygous variant at the splice acceptor site in *MED12* [NM_005120.3: c.4416‐1 T>C] was identified and classified as likely pathogenic during the reanalysis (Figure 3, Table S1). Although this variant was also detected in the initial analysis, the clinical phenotype, including a diaphragmatic hernia, did not align with any previously known MED12‐associated disorders, resulting in suspicion but not a definitive genetic diagnosis. Upon reanalysis, the *MED12* variant (LOEUF score: 0.071) was evaluated with particular attention to the LOEUF score. Thereafter, the Human Gene Mutation Database showed a case report linking Hardikar syndrome, a rare X‐linked dominant disorder caused by *MED12* mutations, to a diaphragmatic hernia (Li et al. 2021). Ultimately, the hemizygous *MED12* variant, with its notably low LOEUF score, illustrates the utility of genetic intolerance scores in prioritizing such genes.

Case #50 involved a preterm birth at 34 weeks of gestation, where meconium peritonitis and multiple malformations were diagnosed (Figure 3B). The proband died during the neonatal period owing to multiple organ failure following subdural hemorrhage. A novel hemizygous missense variant of *CCDC22* [NM_014008.5: c.49A>C (p.Thr17Pro)] was classified as a VUS (PM2 and PM5) in the initial analysis. However, the reanalysis focused on the LOEUF score, especially noting the low score of *CCDC22* (LOEUF score: 0.123). CADD (a protein function prediction software program) predicted that this variant was deleterious (PHRED 29.7) (Table). *CCDC22* is associated with Ritscher–Schinzel syndrome type 2, which shows an XLR inheritance pattern (Kolanczyk et al. 2015). SpliceAI was used to evaluate the effect on splicing because the codon containing this missense variant spanned two exons. Pangolin and MaxEntScan also predicted splice loss (Table). In addition, the N terminus of *CCDC22* constitutes an important functional domain (Starokadomskyy et al. 2013), with a few reports of benign variants documented in ClinVar. The variant was reclassified as likely pathogenic (PM1, PM2, PM5, and PP3) (Figure 3B).

### 3.4 Step 2: Evaluating Variants, Including Intron, for Undiagnosed Cases With Phenotypes Suggestive of Specific Causative Genes Using SpliceAI

We assessed all variants in exon, splice site, and intron of undiagnosed cases of hydrops fetalis and pleural effusion (18 cases), skeletal disorders (8 cases), Joubert syndrome (1 case), and Meckel syndrome (2 cases) (Figure 1). Case #119 was clinically diagnosed with Meckel syndrome, an autosomal recessive disorder; however, the initial analysis identified a maternally inherited stop‐gain variant in *TMEM67* [NM_153704.6: c.2440G>T(p.Glu814*)]. Variants including the intronic region were evaluated using a gene list associated with Meckel syndrome in this step, leading to the identification of an intronic variant in *TMEM67* [NM_153704.6, c.2100 + 3A>G]. A splice donor loss was predicted for this variant by SpliceAI (delta score; 0.38, position; −3 bp), indicating potential intron retention. MaxEntScan also indicated a disrupted conventional splice donor site (Table 1). Thus, this intron variant of *TMEM67* was classified as likely pathogenic. The proband harbored pathogenic and likely pathogenic variants of *TMEM67* in a compound heterozygous manner (Figure 3C).

### 3.5 Step 3: Evaluating Variants, Including Introns, Among all Genes With Low LOEUF Scores Using SpliceAI for Undiagnosed Cases

We assessed all variants in exons, splice sites, and introns of genes with LOEUF scores ≤ 0.35 using SpliceAI for 74 cases that remained undiagnosed even after Steps 1 and 2 (Figure 1). In case #22, intrauterine fetal death was confirmed at 30 weeks of gestation. The SNP array revealed a normal male karyotype. Hydrops fetalis, mild cerebellar hypoplasia, polyhydramnios, and spina bifida were observed in the proband (Figure 4A,B). A gene panel designed for fetal hydrops was used to probe the WES data in Step 2; however, no disease‐causing variants were identified. The reanalysis of all genes with low LOEUF scores led to the identification of a novel de novo hemizygous intronic variant in *CASK* (NM_003688.3, c.2506‐6A>G). This variant (with a LOEUF score of 0.073 for *CASK*) was predicted to cause a potential splice acceptor loss and a splice acceptor gain by SpliceAI, indicating partial intron retention for the variant (Table 1, Figure 4C). RNA sequencing revealed that this intronic variant added a 5‐bp extension to the intronic sequence, leading to aberrant splicing (Figure 4C,D). Some of the symptoms of the affected fetus (cerebellar hypoplasia and polyhydramnios) can be attributed to this variant; thus, it was classified as a pathogenic variant. Mutations in *CASK* cause mental retardation and microcephaly with pontine and cerebellar hypoplasia (MICPCH) syndrome, an XLD disorder. This case involved fetal death, making it difficult to assess detailed clinical symptoms. Finally, by expanding the variant filtering to include intronic regions with the incorporation of the LOEUF score annotation, the reanalysis led to a genetic diagnosis.

### 3.6 X‐Linked Disease‐Causing Genes Exhibit High Intolerance

**FIGURE 5:**
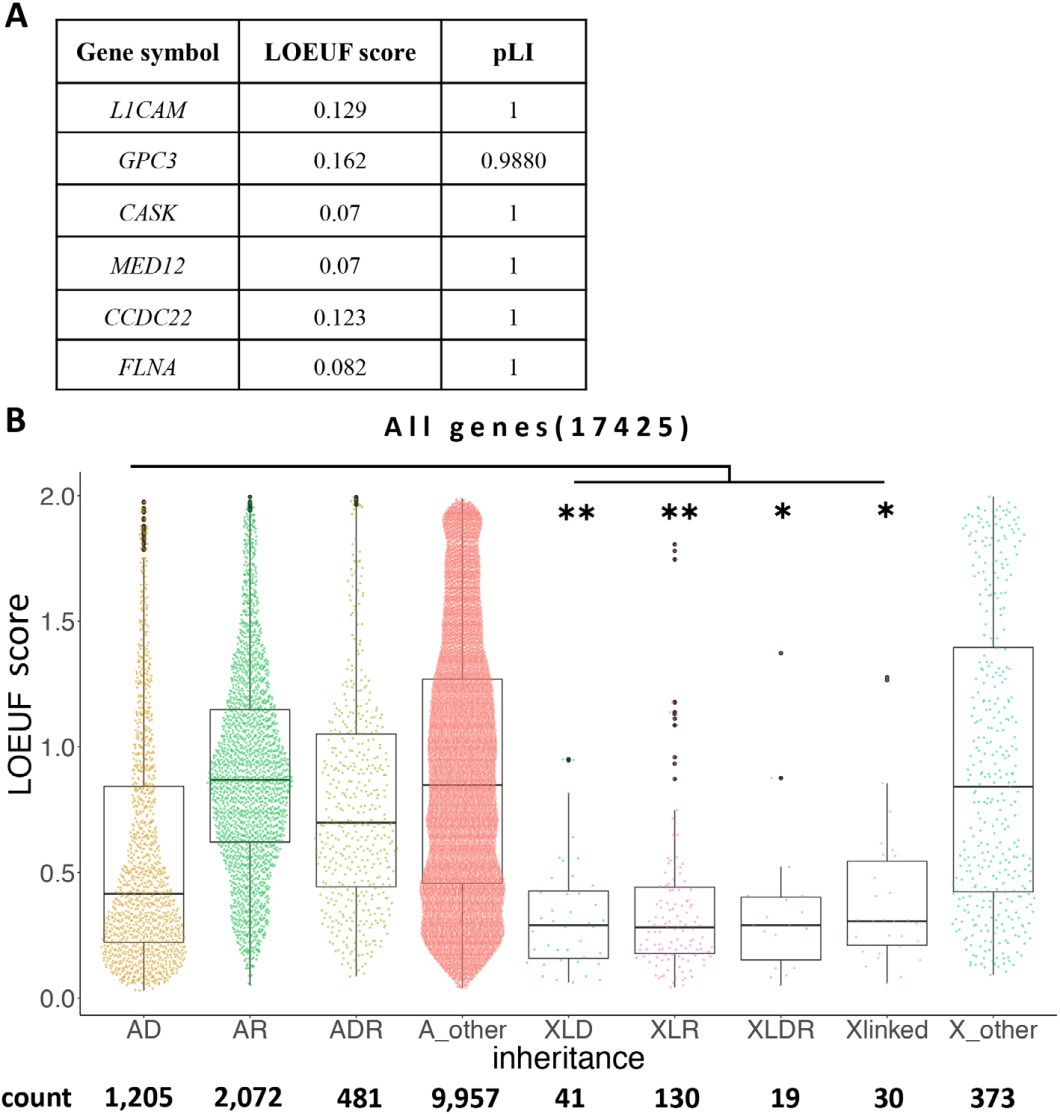
Comparison across all OMIM‐registered genes. (A) List of the LOEUF score of genes on chromosome X identified in our study. (B) Comparison across all OMIM‐registered genes. *p < 0.001. **p < 0.001. Abbreviations: A_other, other genes of the autosome; AD, autosomal dominant; ADR, autosomal dominant and recessive; AR, autosomal recessive; LOEUF, loss‐of‐function observed/expected upper bound fraction; X_other, other genes on chromosome X; XLD, X‐linked dominant; XLR, X‐linked recessive; XLDR, X‐linked dominant and recessive.

All X‐linked disorder genes displayed LOEUF scores of < 0.2 in the present study, indicating their potential to identify candidate X‐linked disorder genes (Figure 5A). Therefore, we determined the LOEUF scores of all disease‐causing genes from the OMIM database and listed the causative genes (see Methods). The LOEUF scores of each X‐linked gene were significantly lower than those of autosomal dominant genes (Figure 5B), suggesting that X‐linked disease‐causing genes are strongly intolerant. The systematic reinterpretation performed in this study focused on low LOEUF scores and facilitated the detection of pathogenic variants, especially on chromosome X.

## 4 Discussion

The initial analysis, including WES and CNV analyses, identified 51 (39.8%) of the 128 congenital anomalies. In the reanalysis, we also evaluated intron variants in the WES data by adding genetic intolerance score annotations, which finally led to four new genetic diagnoses (5.2%, 4/77 cases, Figure 1). Our results showed that several pathogenic variants were found in genes with low LOEUF scores, particularly in those associated with X‐linked disorders (Figures 3 and 5). This finding suggests that genetic intolerance scores can assist in focusing on such genes. Furthermore, the analysis of intronic regions in WES data was found to be beneficial for diagnosis. These methods are valuable tools for efficiently evaluating variants in fetal diseases, which are clinically challenging to assess.

Recent advancements have demonstrated the efficacy of incorporating genetic intolerance scores, such as the LOEUF score, into genome analysis (Seaby et al. 2023). Additionally, emerging evidence supports the inclusion of intronic regions in WES analysis to enhance the diagnosis of monogenic diseases (Liu et al. 2021; Li et al. 2023). Therefore, in this study, we employed distinct approaches for WES and incorporated advanced genetic intolerance scores and splice prediction tools in the reanalysis. In Step 1, the LOEUF score guided a reanalysis that incorporated information from the latest literature, ultimately leading to a conclusive diagnosis for two X‐linked diseases (Cases #23 and #50) (Gjerulfsen et al. 2021; Li et al. 2021). In Case #23, SpliceAI predicted that the *MED12* splice donor variant causes acceptor gain, leading to LOF (Figure 3A). In Case #50, a novel *CCDC22* variant (c.49A>C) had a higher CADD score than a reported pathogenic variant (c.49A>G), which disrupted NF‐κB activation through splicing abnormalities. The current variant also predicts donor loss, likely causing intron retention and expression reduction, potentially leading to NF‐κB dysfunction (Table 1, Figure 3B). The LOEUF scores aided gene identification in both cases. In Step 2, reanalysis using SpliceAI, which included intronic regions previously excluded from variant interpretation, led to the diagnosis of Meckel syndrome (Case #119) that was initially missed. SpliceAI and MaxEntScan together indicated splice donor loss, supporting the diagnosis and underscoring the importance of analyzing intronic regions in WES to identify disease‐causing genes.

This study demonstrated that the analysis of intronic variants in genes with low LOEUF scores can aid in identifying pathogenic variants in complex phenotypes (wherein the causative gene is difficult to predict). In Step 3, this approach detected a pathogenic intronic variant in *CASK* (Case #22). Rodríguez‐García et al. (2023) also reported the same variant in a 9‐year‐old boy, causing an intronic addition in 60% of transcripts, whereas 20% retained normal splicing. However, the proband in this study was born at full term without abnormalities, suggesting the involvement of additional factors, as fetal death is not directly linked to CASK disorders. In Case #22, a *CALM1* intronic variant (LOEUF score: 0.277) was identified, with SpliceAI predicting donor gain and RNA‐seq confirming abnormal transcripts (Figure S1). *CASK* expression, which relies on calmodulin, peaks early in fetal cerebellar development, whereas *CALM1* expression increases later (Kang et al. 2011, Figure S2), suggesting their importance in cerebellar development. Pathogenic *CALM1* variants are associated with life‐threatening arrhythmias and sudden death in young individuals, although no arrhythmia history was reported in the father carrying this variant (Crotti et al. 2013). Furthermore, *CASK* knockout induces heart failure via CaMKII activation (Mustroph et al. 2021), highlighting the critical roles of calmodulin‐related genes in cardiac function. Future studies should explore the relationship between *CASK* and *CALM1* and their association with fetal abnormalities and fetal death.

Notably, genes associated with XL disorders (especially in the current case set) consistently exhibited low LOEUF scores after reanalysis. An overarching survey across all OMIM‐registered genes revealed that XL disorder genes always present with significantly lower LOEUF scores (Figure 5), thereby emphasizing potential diagnostic goldmines with low LOEUF‐scoring non‐OMIM genes.

Nevertheless, the present study had some limitations. In over 50% of cases, WES could not clarify the etiology of morphological abnormalities, which may reflect a combination of nonmonogenic etiologies and the current limitations of WES in detecting all potential genetic contributors. The significant mortality rate among the patients added a layer of complexity to variant interpretation owing to the absence of clinical information. Moreover, WES data evaluation was confined to the intron variants proximal to exons, signaling a shift toward whole genome sequencing for holistic variant assessment in subsequent endeavors. Another significant challenge was the poor quality of RNA obtained from stillbirths and terminated pregnancies; therefore, transcriptional analysis for cases other than Case #22 could not be conducted appropriately.

Integrating Steps 2 and 3 into the initial analysis would require optimizing the procedure for efficiency. Because applying these steps to all the cases is inefficient, they are currently optional components of the initial analysis pipeline. Nevertheless, Step 3—which involves evaluating all intronic variants with low LOEUF scores across all samples—has the potential to identify novel causative genes. Therefore, we plan to incorporate Step 3 into the initial analysis pipeline in the future, a nonoptional component to enhance its capabilities.

## 5 Conclusion

This study highlights the potential of an integrative approach for WES data reanalysis. Incorporating the analysis of intronic variants and leveraging genetic intolerance scores enhances the diagnostic yield for congenital anomalies.

## Author Contributions

K.T. and K.H. conceptualized the study and wrote the draft version of the paper. K.T., F.H., Y.O., A.H., H.O.K., S.A., O.M., T.K., K.N., and K.O. conducted the analysis. Variants were evaluated by discussing various clinical information with K.F., S.W., K.O., Y.I., and H.S. All authors reviewed and completed the paper.

## Conflicts of Interest

The authors declare no conflicts of interest.

## Supporting information


**Figure S1.** (A) Pedigree of this case and each genotype. (B) Overview of the intronic variants that cause aberrant splicing. (C) Reads indicating aberrant transcripts from RNA‐seq data.
**Figure S2.** Horizontal axis: gestational age of the fetus, vertical axis: relative gene expression level when 10–12 weeks is used as a control. The expression in gray does not change, compared with that during 10–12 weeks, and is considered equivalent to that of the control for convenience; however, the expression in red is significantly upregulated, whereas that in blue is significantly downregulated.

Tables S1–S4.

## Data Availability

Research data are not shared.
