# Supplementary figures and images for: Approaches to Evaluate Whole Exome Sequencing Data That Incorporate Genetic Intolerance Scores for Congenital Anomalies, Including Intronic Regions Adjacent to Exons
Source: Mol Genet Genomic Med. 2025 Mar 13;13(3):e70092. doi: 10.1002/mgg3.70092 (PMC11904091; doi:10.1002/mgg3.70092)

A

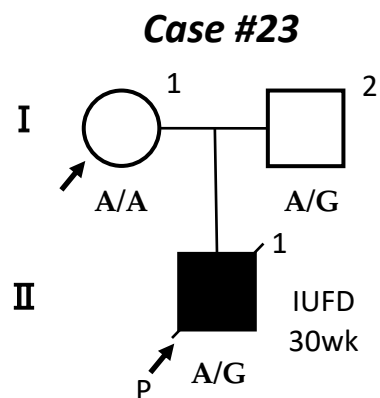

***CALM1*:NM\_006888.6:c.34+126A>G**  
(ACMG classification; PM2 and PP3, VUS )

B

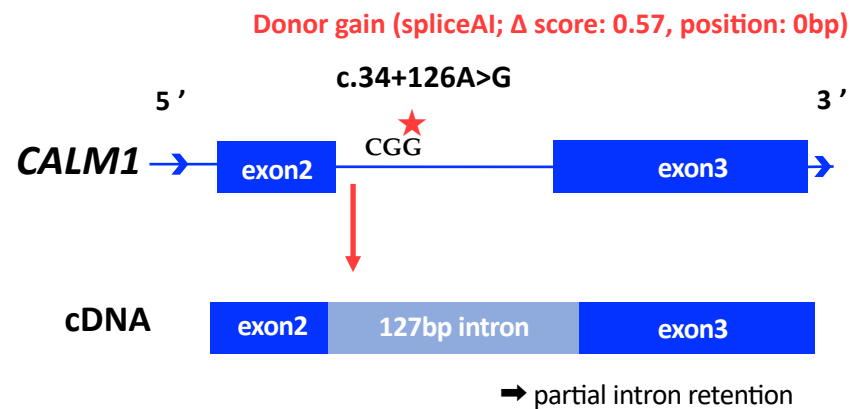

C

**WT/c.34+126A>G**

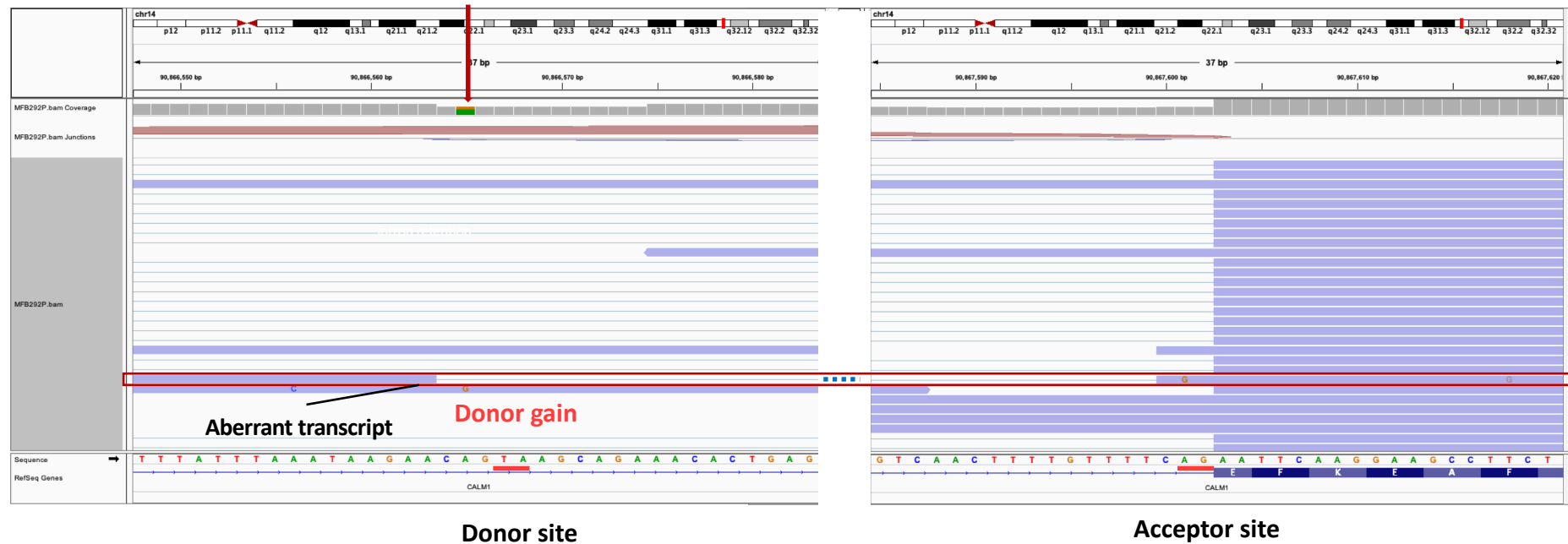

**sFigure1**

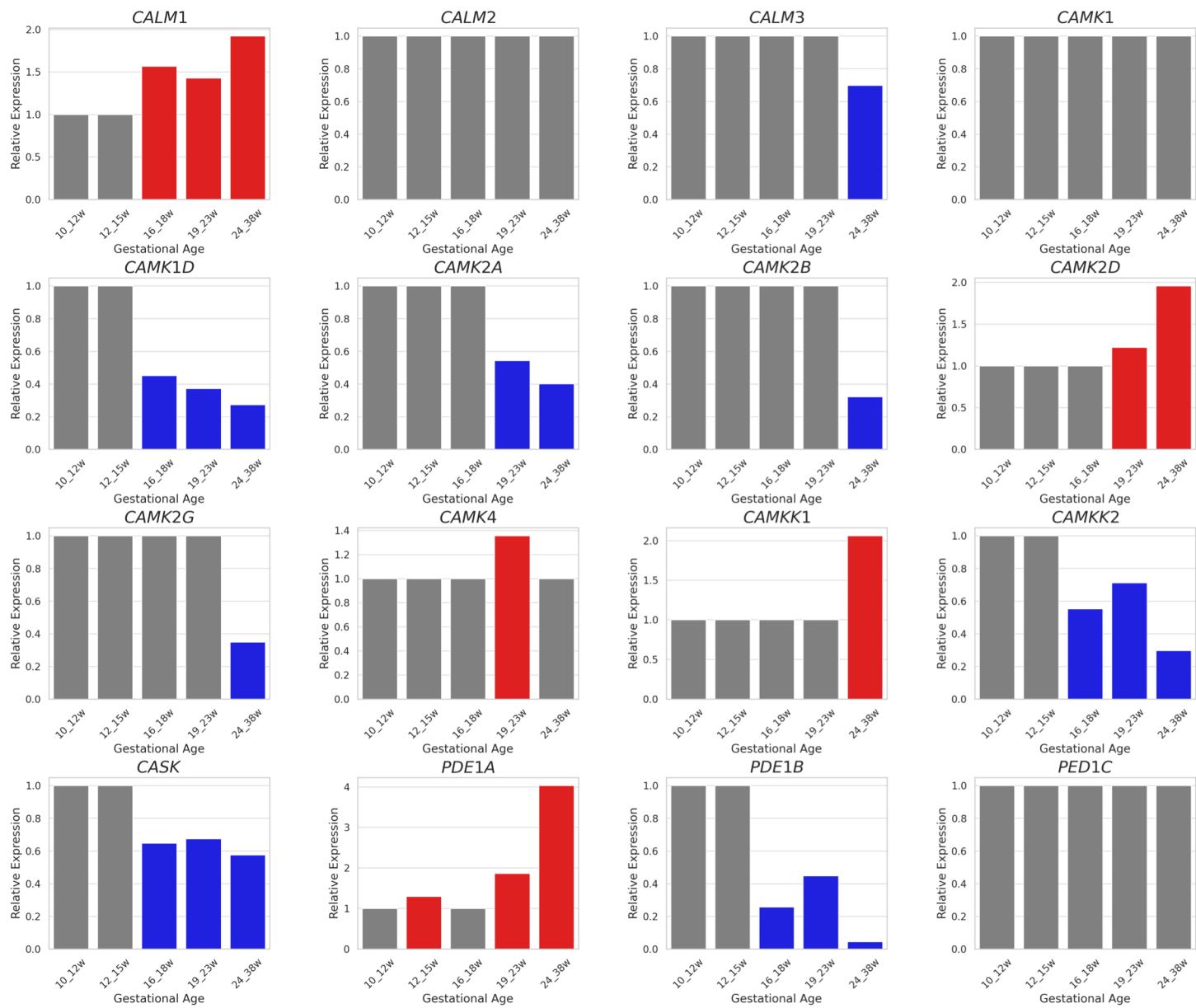

Supplement: Supplementary file 1 — Figure S1. (A) Pedigree of this case and each genotype. (B) Overview of the intronic variants that cause aberrant splicing. (C) Reads indicating aberrant transcripts from RNA‐seq data. Figure S2. Horizontal axis: gestational age of the fetus, vertical axis: relative gene expression level when 10–12 weeks is used as a control. The expression in gray does not change, compared with that during 10–12 weeks, and is considered equivalent to that of the control for convenience; however, the expression in red is significantly upregulated, whereas that in blue is significantly downregulated. [file MGG3-13-e70092-s001.pdf]
